# A Multidisciplinary Fingerprinting Approach for Authenticity and Geographical Traceability of Portuguese Wines

**DOI:** 10.3390/foods10051044

**Published:** 2021-05-11

**Authors:** Rui J. S. Teixeira, Sónia Gomes, Vitorino Malheiro, Leonor Pereira, José R. Fernandes, Alexandra Mendes-Ferreira, Maria E. P. Gomes, Paula Martins-Lopes

**Affiliations:** 1 School of Life Science and Environment, University of Trás-os-Montes e Alto Douro, UTAD, Quinta de Prados, 5000-801 Vila Real, Portugal; rteixeir@utad.pt (R.J.S.T.); sgomes@utad.pt (S.G.); vitorinoltmalheiro@hotmail.com (V.M.); leopereira@utad.pt (L.P.); jraf@utad.pt (J.R.F.); anamf@utad.pt (A.M.-F.); mgomes@utad.pt (M.E.P.G.); 2 Department of Geology and Pole of the Geosciences Centre (CGeo), University of Trás-os-Montes e Alto Douro, 5000-801 Vila Real, Portugal; 3 Faculty of Sciences, BioISI-Biosystems & Integrative Sciences Institute, University of Lisboa, 1749-016 Lisboa, Portugal; 4 CQVR and Department of Physics, University of Trás-os-Montes e Alto Douro, 5000-801 Vila Real, Portugal; 5 WM&B-Laboratory of Wine Microbiology & Biotechnology, Department of Biology and Environment, University of Trás-os-Montes e Alto Douro, 5000-801 Vila Real, Portugal

**Keywords:** *Vitis vinifera* L., wine authenticity, high-resolution melting, geographical provenance, Sr and Pb isotopic data, Alvarinho, Douro

## Abstract

The interest in developing reliable wine authenticity schemes is a hot-topic, especially for wines with recognized added-value. In order to accomplish this goal, two dimensions need to be considered: the grapevine variety determination and the geographical provenance. The aim of this study was to develop a multidisciplinary approach applicable to wines from the sub region Melgaço and Monção of the demarcated Vinho Verde region and from the demarcated Douro region. The proposed scheme consists on the use of DNA-based assays to detect Single Nucleotide Polymorphisms (SNPs) on three genes of the anthocyanin pathway (*UFGT*, *F3H* and *LDOX*) coupled with High-resolution melting (HRM) analysis aiming the varietal identification. The Alvarinho wines revealed to have the same haplotype using this marker set, demonstrating its applicability for genetic identification. In addition, to assess their geographical provenance, a multi-elemental approach using Sr and Pb isotopic ratios of wine, soil and bedrock samples was used. The isotopic data suggest a relation between Sr and Pb uptake by vine roots and soil’s texture and clay content, rather than with the whole rock’s isotopic ratios, but also highlights the potential of a discriminating method based on the combination of selected isotopic signatures.

## 1. Introduction

Global food products and beverages markets have increased the need to establish reliable and quick authenticity systems so they can be tracked, throughout the production and/or commercial chain, to its origin. The development of such a system has the advantage of guaranteeing consumers that the purchased products are in accordance to their expectations and avoid the enormous economic damages caused by forgeries and even health risks, due to the presence of dangerous compounds and contaminants in the counterfeited food products [1].

To assure the consumers needs’ and requirements in such differentiated food products, several certification labels (e.g., protected designation of origins—PDO and protected geographical indications—PGI labels) have been regulated by the European Union with different denominations (Regulations Nos. 509/2006, 510/2006 and 1898/2006) [2,3,4]. The wine sector has a long tradition in the protection of differentiated wines, and ever since the 1700s a series of appellations were created: in 1716, the Chianti classification system in Italy; in 1730, the Tokaj-Hegyalja classified the Hungarian vineyards according to soil, sun exposition and *Botrytis cinerea* tolerance and, in 1756, the first region with denomination of origin (DO), Porto, was created in Portugal. From then on, several appellation and wine control systems have been implemented worldwide. These denominations are defined based on both the terroir features (soil and grapevine-associated microbiota, climate, wine making process, agronomical practices, etc.) and in the grapevine varieties used to produce such wines, giving them unique characteristics that can differentiate those wines in relation to other, having important economic returns [5,6]. However, due to their interesting market values, they are often subject to fraudulent practices, which require the establishment of a reliable control system so these can be detected and avoided.

The need to control the denomination of origin requires the implementation of a robust authenticity system, aiming to control both the grapevine variety and the geographical origin used in a given wine [7,8,9]. Since the edapho-climatic characteristics influence the wine chemical composition, their direct application in grapevine varietal discrimination is not recommended. Instead, DNA-based marker systems have been successfully applied to discriminate wine varietal assignment, using Single Sequence Repeat markers—SSR [10,11,12,13] and Single Nucleotide Polymorphism markers—SNP [9,14,15], but they are not suitable for wine geographical determination. Therefore, several methodological approaches to identify the geographical provenance of processed beverages, such as wine, have been proposed throughout the years, but those centered on the wine chemical compounds, namely in their anthocyanin composition, and in the geochemistry of heavy (Sr, Pb) and light (H, C, O, N, B) stable isotopes, sometimes combined with multi-elemental analysis and chemometrics, have demonstrated to be the most accurate [16,17,18,19,20,21,22,23,24]. However, it should be noted that climatic variations preferentially affect the isotopic compositions of light elements, in opposition to those of radiogenic heavy elements, allowing advantageous direct correlations between Sr and Pb isotopic compositions of the geological/pedological substrata from production areas and those of the respective agricultural products [17,25].

**Figure 1:**
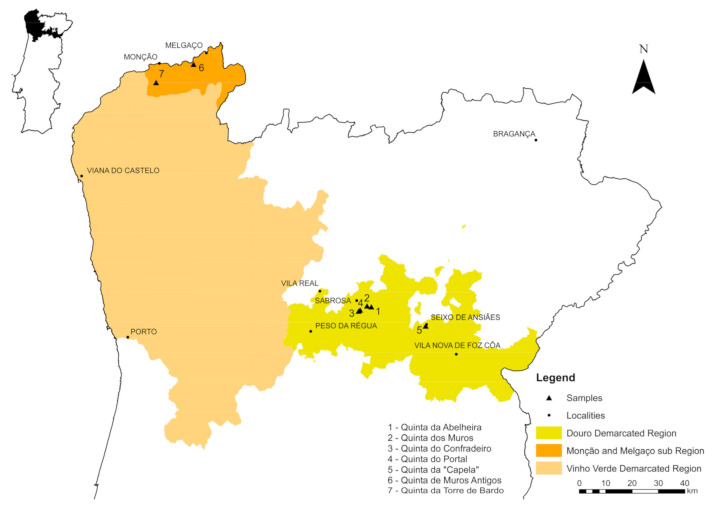
Map of northern Portugal showing the locations of selected vineyards under study.

In Portugal, there are contrasting features between the geological and soil environments of the Melgaço and Monção sub region [26,27] of the Vinho Verde demarcated region (Figure 1), which extends across the northwest of Portugal with vineyards on granitic derived soils, and those from the valley vineyards of the Douro demarcated region (Figure 1), mainly emplaced in metasedimentary derived soils. In this paper we report the results of Sr and Pb isotopic determinations and particle size analyses performed in rock and soil samples from the aforementioned areas, in order to identify possible unambiguous traceability scientific parameters that can be helpful to solve wine authenticity and geographical origin assessment issues. As no single method is able to accurately identify both dimensions, in this study the varietal discrimination was assessed using a molecular marker approach, based on varietal SNP information applied to High-resolution melting—HRM analysis, allowing the application of an integrated authenticity system to the aforementioned areas.

### Geological Setting

In northern Portugal, two-mica granites and biotite-rich granites intruded neoproterozoic to devonian metasedimentary rocks during the Variscan orogeny [28,29]. In the sub region Melgaço and Monção of the Vinho Verde demarcated region (Figure 1), two distinct granites are found: (a) at Melgaço, a syn-D_3_ medium-grained two-mica granite, yielding a strong penetrative foliation developed under ductile deformation conditions; (b) at Monção, a post-D_3_ coarse- to medium-grained porphyritic biotite granite, without major evidences of ductile deformation, but affected by some fracturing. The Douro demarcated region is essentially characterized by metasediments of neoproterozoic-cambrian age, which, in the area of interest of this study, are subdivided in the following formations: (a) Rio Pinhão Formation, formed by a succession of thick metagreywackes interbedded with phyllites and some levels of microconglomerates; (b) Pinhão Formation, composed by chloritic phyllites, quartzo-chloritic phyllites and metagreywackes; (c) Desejosa Formation, typically consisting of millimetre- to centimetre-scale alternations of parallel-laminated metapelites and fine-grained psammites, resulting in a striped appearance, that makes it very distinct from the two previous formations [30,31]. Geochemically, there are also differences between Desejosa and Pinhão Formations, since the first one shows higher SiO_2_ and lower Al_2_O_3_ average contents than the latter [32]. Although no modal compositions have been determined, those geochemical differences are certainly accompanied by a quartz enrichment and phyllosilicates impoverishment in Desejosa Formation. However, in the Douro demarcated region some intrusions of syn-D_3_ granites also occur. An example is given by the medium-grained slightly porphyritic two-mica granite that appears in Seixo de Ansiães area, which yields an internal northwest—southeast magmatic foliation, concordant with the structure of the host metasediments [33,34].

## 2. Materials and Methods

### 2.1. Sampling

In this study, different samples of wine (11), soil (11) and bedrock (11) were collected from seven vineyards of the two demarcated wine-producing regions: (1) Monção and Melgaço sub region and (2) Douro region (Table 1 and Figure 1). The soil samples were collected from a depth layer of 10–20 cm, whereas the bedrock samples were collected in the same row of the corresponding grapevine samples. Bedrock and soil samples were crushed in a jaw crusher and grinded in an agate mill at the Department of Geology of University of Trás-os-Montes e Alto Douro. The sampled grapevine varieties were Alvarinho (white grapevine), Moscatel Galego (white grapevine), Touriga Franca (red grapevine) and Touriga Nacional (red grapevine) (Table 1).

The grapevine varieties Touriga Franca and Touriga Nacional at Quinta do Portal and Quinta do Confradeiro vineyards (both implanted in the Desejosa Formation) and Alvarinho and Touriga Nacional at Quinta da Abelheira vineyards (both implanted in the Rio Pinhão Formation) were used as a control in order to assess a possible influence of metabolic processes from plants on Sr and Pb isotopic signatures’ fractionation of wines produced in a particular geological/soil environment.

### 2.2. Vinification of White and Red Grape Varieties

All wines resulted from monovarietal micro-vinifications to avoid possible multi-isotopic variability (Table 1). Grapes were crushed, destemmed and pressed separately. Sulfur dioxide (SO_2_; 60 mg/L) was added to both red and white musts. White grape-must was clarified by cold settling at 4 °C for 36 h. Red grape-musts (along with grape skins) and white grape-must were transferred to 2 L containers filled to 2/3 of their volume and fitted with a rubber cork fixed with an air locker. All grape-musts were supplemented with 1 g/L of di-ammonium phosphate (DAP). Alcoholic fermentations were conducted at 17 °C (white grape-musts) and at 25 °C (red grape-musts) by the commercial yeast strain *S. cerevisiae* QA23 inoculated at an initial cell count of 10^6^ CFU/mL. The vinifications were daily monitored by weight loss, as an estimate of CO_2_ production, and were allowed to proceed until no further weight loss was observed. After the end of alcoholic fermentation, the wines were racked off the lees and cold settled at 4 °C. Before bottling, SO_2_was added to wines to adjust free sulfur dioxide content to 35 mg/L and to 50 mg/L, for red and white grape-must, respectively. The standard physico-chemical analyses were performed prior to and at the end of alcoholic fermentation, according to the Organisation International de la Vigne et du Vin (OIV) official methods [35]. The oenological characteristics of wines are summarized in Table S1.

### 2.3. Genotyping Using High-Resolution DNA Melting Assay

Young leaves of Alvarinho, Touriga Franca and Touriga Nacional grapevine varieties were collected from different vineyards and immediately frozen in liquid nitrogen, in order to be used as a reference material to develop the HRM assays. Total genomic DNA was extracted from the frozen young leaf samples using the CTAB method [36]. DNA samples were extracted from Alvarinho, Touriga Franca and Touriga Nacional wine samples, belonging to the Monção and Melgaço sub region and to the Douro region (Table 1), using the protocol described by Pereira et al. [12]. The purity and quantity of all DNA samples was estimated by Nanodrop™ 1000 Spectrophotometer (Thermo Fisher Scientific, Wilmington, DE, USA) measurements and checked for integrity by electrophoresis on a 0.8% agarose in 1X TAE (Tris-acetate-EDTA). Sequences of the different Alvarinho leaf samples were checked (http://www.stabvida.com, accessed on 1 April 2021) using three previously studied genes: UDP-glucose: *flavonoid 3-O-glucosyltransferase* (*UFGT*) [8,33], *flavanone 3-hydroxylase* (*F3H*) and *leucoanthocyanidin dioxygenase* (*LDOX*) [34].

PCR reactions and HRM analysis were performed in a StepOne™ Real-Time PCR System (Applied Biosystems, Foster, CA, USA) according to Pereira et al. [9,37] and Gomes et al. [38]. To validate the results of HRM profiles, the post-HRM products were purified using illustraExoProStar 1-Step Kit (GE Healthcare Life Sciences, Foster, CA, USA) and the purified PCR products were sequenced in both directions, with the same PCR primers, using STAB VIDA services (http://www.stabvida.com, accessed on 1 April 2021). The CodonCode Aligner 4.0.4 (CodonCode Corporation, Centerville, MA, USA) was used to generate consensus sequences, and sequence alignment was performed using Geneiousv5.6.4. The HRM data was analysed using the High-Resolution Melt Software v3.0.1 (Applied Biosystems, Foster, CA, USA). After normalization and determining the temperature shift, the different melt curves of the several plots were generated.

### 2.4. Isotopic Analysis

Strontium isotopic analyses of bedrock, soil and wine samples were obtained at the Laboratory of Isotope Geology of the University of Aveiro (LGI-UA), Portugal, following the methodology described by Costa et al. [39], with some adaptations in the case of wine samples. In a first stage, bedrock and soil powders were dissolved in a hydrofluoric acid (HF) and nitric acid (HNO_3_) mixture in pressurized teflon capsules (Savillex^®^) on a heating plate for three days, then dried and taken up in hydrochloric acid (HCl) for chemical separation. The wines were also digested on a heating plate, but through a reflux system with HNO_3_ and hydrogen peroxide (H_2_O_2_) in successive stages. After the total digestion of the different samples, the resulting solutions were evaporated and the residues were dissolved with HCl (6.2 N) and dried again. Cation exchange resin (BioradAG50W) in quartz glass columns was then used to separate the Sr fraction by ion chromatography. All reagents used in the preparation of the samples were purified by bi-distillation. The ultrapure water (18.2 MΩ.cm) was obtained by a Milli-Q Element system^®^ (Millipore, Burlington, MA, USA). ^87^Sr/^86^Sr isotopic ratios were measured on a VG Sector 54™ mass spectrometer (VG Instruments Group, West Sussex, UK) operating in dynamic mode. The different Sr samples were deposited in a Ta filament with phosphoric acid (H_3_PO_4_) (0.5 N) and by maintaining a 1–2 V 88Sr beam for 50–100 cycles, an internal precision of 20 ppm (at 2σ level) on ^87^Sr/^86^Sr was consistently achieved. Fractionation was corrected with an exponential law relative to ^86^Sr/^88^Sr = 0.1194. During the measuring campaign, the value determined for the Standard Reference Material^®^ (SRM) 987 was ^87^Sr/^86^Sr = 0.710256 ± 0.000005 (2σ; N = 23) and 0.710271 ± 0.000013 (2σ; N = 16). Analytical blanks for Sr are lower than 250 pg.

The Pb isotopic analyses of bedrock and wine samples were performed at the Geochronology and Isotope Geochemistry-SGIker Facility of the Universidad del País Vasco UPV/EHU (Spain). Samples were prepared in PP Class-A (ISO-5) laminar flow bench within an ISO-7 clean lab. Geological and wine samples were digested following procedures adapted from Marchionni et al. [19]. Lead was isolated with the selective extraction material Sr.Spec, following the chromatographic procedures miniaturized from Deniel and Pin [40]. The purified Pb samples were dissolved in 1.5 mL of 0.32 N HNO_3_. For geological samples, the solutions were conveniently diluted to obtain a concentration of about 200 ng of about Pb/g solution. The samples were introduced as wet aerosols into a Neptune™ MC-ICP-MS (Thermo Fisher Scientific, Bremen, Germany) using an ESI 100 µL/min PFA nebulizer (Elemental Scientific Instruments, Omaha, NE, USA) and a dual cyclon-ic-Scott double pass spray chamber (Elemental Scientific Instruments, Omaha, NE, USA). Lead amounts from wine samples were lower than 100 ng/g solution. Therefore, wine samples were diluted to a 20 ng/g solution and introduced as dry aerosols with an ESI 50 µL/min PFA nebulizer (Elemental Scientific Instruments, Omaha, NE, USA) and an ESI Apex-IR desolvating unit (Elemental Scientific Instruments, Omaha, NE, USA). The spectrometric measurement comprised a static multi-collection routine of 10^5^ cycles with an integration time of 8 s per cycle. Spectrometric (chemical + electronic) blanks were subtracted with the On-Peak-Zeroes routine, measuring a blank 0.32 N HNO_3_ solution before each sample for 60 s. Instrumental mass bias was corrected online after the addition of a proportional amount of a solution of the National Bureau of Standards^®^ (NBS) 997 thallium certified reference material and using ^205^Tl/^203^Tl = 2.3889 according to Thirlwall et al. [41]. The accuracy and reproducibility of the method was verified by periodic determinations, under the same instrumental conditions, of the certified reference material NBS 981. The average ratio, at 2σ level, of 4 determinations during the same analytical sessions were: ^206^Pb/^204^Pb = 19.9442 ± 0.0022; ^207^Pb/^204^Pb = 15.5016 ± 0.0011; ^208^Pb/^204^Pb = 36.7317 ± 0.0056; ^208^Pb/^206^Pb = 2.16781 ± 0.00010; ^207^Pb/^206^Pb = 0.91486 ± 0.00006.

### 2.5. Particle Size Analysis

The particle size analysis of selected metasedimentary and granitic soils was carried out on full robotized equipment from Skalar Analytical (Breda, The Netherlands) at the Laboratory of Soils and Plants—Joaquim Quelhas dos Santos of University of Trás-os-Montes e Alto Douro, Portugal, following the International Organization for Standardization (ISO) 11277 method by the sedimentation/pipetting option.

## 3. Results and Discussion

### 3.1. Varietal Discrimination—Genotyping by High-Resolution DNA Melting Analysis

**Figure 2:**
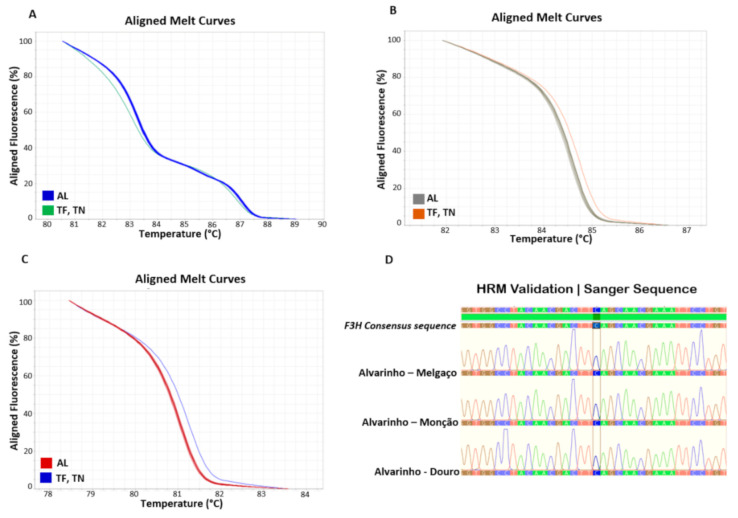
High-resolution DNA melting curve analysis for Alvarinho varieties, with different geographical provenance, analysed with three genes belonging to the anthocyanin pathway: (**A**) UDP glucose–flavonoid 3-O-glucosyl-transferase (UFGT); (**B**) Flavanone 3-hydroxylase (F3H) and (**C**) Leucoanthocyanidin dioxygenase (LDOX). The HRM profiles, based on varietal gene sequence, revealing only one haplotype/variant. Different Alvarinho clones were tested to validate the technique for varietal fingerprinting, and the results show an unique melting curve, accordingly to the nucleotide sequence (**D**). AL—Alvarinho, TF—Touriga Franca, TN—Touriga Nacional, HRM—High-resolution melting.

To confirm the varietal composition of Alvarinho, Touriga Franca and Touriga Nacional wine samples, a DNA–based method was tested, using high–resolution DNA melting analysis to detect variants/haplotypes, based on specific SNPs detected within the nucleotide sequence of three genes previously studied, UFGT, F3H and LDOX [9,38]. To evidence that this technology can be used for varietal identification, the wines obtained from Monção and Melgaço sub region and Douro region were tested, and the HRM profile based on the gene sequence of UFGT (Figure 2A), F3H (Figure 2B), and LDOX (Figure 2C), revealed that only one haplotype was present, with an unique melting curve, demonstrating the same genotype within the given variety. The unique sequence obtained among the different Alvarinho accessions (e.g., F3H sequence; Figure 2D), revealed that these markers can be used as a reliable method for varietal identification in wine samples, as was previously reported by Pereira et al. [9] in other grapevine varieties.

The grapevine varietal identification in wine samples is extremely relevant [37,38,42]. Single Sequence Repeats (SSRs) molecular markers have been applied in wine samples for varietal composition identification [10,11,12,13], however, some difficulties have been reported, mainly due to the quality and quantity of DNA recovered from such samples. Santos et al. [42] have suggested that smaller SSR loci should be used for such purpose. The resource to SNP markers, as an alternative to SSRs markers, has demonstrated to be a good choice, since they are highly stable and repeatable, with a high discriminating power [43], with excellent results in wine samples [9,14]. Currently, HRM represents one of the simplest method used in high throughout put genotyping. Considering wine authenticity, HRM can detect small nucleotide variants within genes belonging to the anthocyanins pathway (Figure 2), with a remarkable precision, overcoming the problems found when using SSR markers. More, HRM assays can be used for grapevine varietal identification throughout the wine production chain, since it is applicable to all sample types (vine–grape–must–wine).

### 3.2. Wine Geographical Provenance

#### 3.2.1. Tracing Wine Origin Based on Sr Isotopic Composition

**Figure 3:**
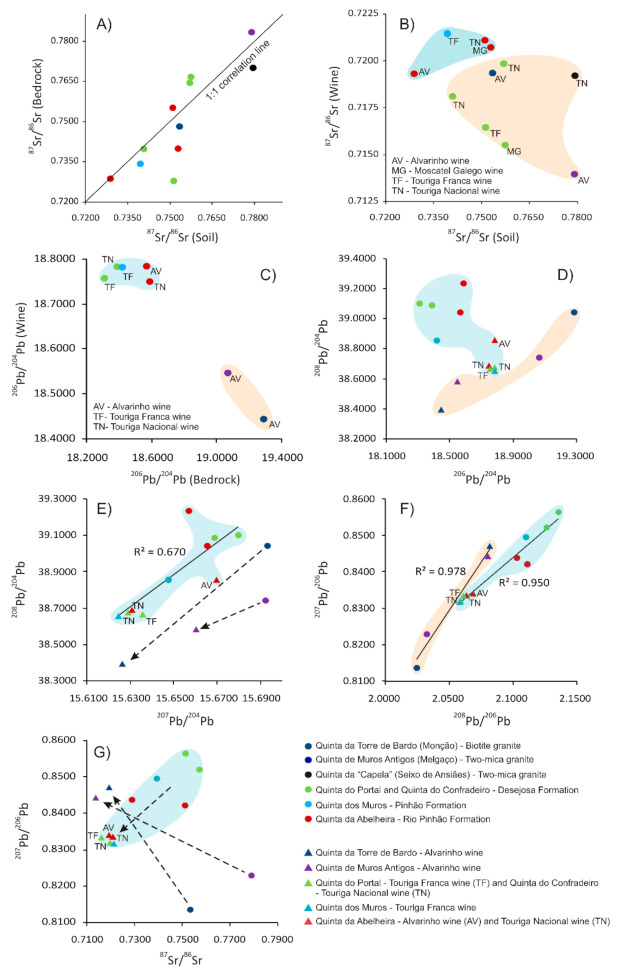
Sr and Pb isotopic ratios in wines, soils and rocks from vineyards located in Vinho Verde and Douro wine producing regions. (**A**) ^87^Sr/^86^Sr ratios in metasedimentary and granitic bedrocks and respective soils; (**B**) ^87^Sr/^86^Sr ratios in Alvarinho, Moscatel Galego, Touriga Franca and Touriga Nacional wines and respective soils; (**C**) ^206^Pb/^204^Pb ratios in Alvarinho, Touriga Franca and Touriga Nacional wines and respective bedrocks; (**D**) ^208^Pb/^204^Pb and ^206^Pb/^204^Pb ratios in Alvarinho, Touriga Franca and Touriga Nacional wines and respective bedrocks; (**E**) ^208^Pb/^204^Pb and ^207^Pb/^204^Pb ratios in Alvarinho, Touriga Franca and Touriga Nacional wines and respective bedrocks; (**F**) ^207^Pb/^206^Pb and ^208^Pb/^206^Pb ratios in Alvarinho, Touriga Franca and Touriga Nacional wines and respective bedrocks; (**G**) ^207^Pb/^206^Pb and ^87^Sr/^86^Sr ratios in Alvarinho, Touriga Franca and Touriga Nacional wines and respective bedrocks/soils.

The values of ^87^Sr/^86^Sr isotopic ratios in whole rocks, bulk soils and wines from vineyards of eleven different locations are reported in Table S2. The bedrock’s ^87^Sr/^86^Sr values from the studied areas range from 0.727938 ± 0.000020 (Desejosa Formation–Quinta do Portal) to 0.783222 ± 0.000020 (two-mica granite–Melgaço), whereas those from soils are within the range of 0.728944 ± 0.000020 (Rio Pinhão Formation–Quinta da Abelheira) and 0.779035 ± 0.000019 (two-mica granite–Melgaço) (Table S2). The variation of ^87^Sr/^86^Sr values in bedrocks and respective soils suggests a strict relationship between them, since rock-soil pairs tend to plot near to the 1:1 correlation line (Figure 3A), fact that is probably conditioned by the incipient soil formation features of the analysed soils, which are classified as leptosols and eutriccambisols, respectively in the metasedimentary and granitic areas from the Douro region, and as umbrisols (humiccambisols) at Melgaço and Monção granitic areas, despite of some human influence in their origin [44]. The largest deviation from 1:1 correlation line occurs in a sample from Desejosa Formation–Quinta do Portal, and probably was due to local differences in the nature of the soil, leading to an ^87^Sr/^86^Sr increase from bedrock to soil (Figure 3A). Furthermore, it is worth noting that the ^87^Sr/^86^Sr values found in the studied metasedimentary rocks from Rio Pinhão, Pinhão and Desejosa Formations and in the two-mica granite from Seixo de Ansiães, and in their respective soils, are higher than those found by Catarino et al. [20] in soil samples from four different vineyards in the Douro region (0.715 ± 0.001 to 0.718 ± 0.0006).

In the studied areas, the wines’ ^87^Sr/^86^Sr values are within the range of 0.713981 ± 0.000024 (Alvarinho–Melgaço) and 0.721478 ± 0.000017 (Touriga Franca–Pinhão Formation) and, generally, there are no significant differences between grape varieties (Table S2), which is agreement with the findings of Kawasaki et al. [45] and Geană et al. [46]. These results are similar to those determined for wines from other Portuguese demarcated regions, such as Dão region [21] (0.713–0.715), Bairrada region (an average of 0.710), Borba region (an average of 0.710), Madeira region (an average of 0.708), Óbidos region (0.708–0.710) and Palmela region (0.708–0.710) [20].

Considering that wines’ ^87^Sr/^86^Sr values have been determined at the same uncertainty level as those of the soil samples, a direct comparison was made to verify their relation (Figure 3B). In this plot, there is a consistent depletion in ^87^Sr/^86^Sr values from bulk soil samples to the respective wines, which is in accordance with the results of Tescione et al. [17] for vineyards from southern Tuscany, Central Italy. On the other hand, wines from vineyards implanted in metasediments from Rio Pinhão and Pinhão Formations generally yield higher ^87^Sr/^86^Sr values than those from Desejosa Formation metasediments and granitic areas (Figure 3B).

Within granitic areas, the wines from Monção and Seixo de Ansiães areas yield higher Sr ratios than that from Melgaço area (Figure 3B). According to Marchionni et al. [18,19], Petrini et al. [47] and Tescione et al. [17] the variation on ^87^Sr/^86^Sr depletion from soil to bioavailable fraction is dependent on the texture and inorganic composition of soils, which play an important role in the release control of the bioavailable fraction to vines, determining specific relations among Sr ratios of bedrock, soil, vine, and wine [17,18,47]. Among other factors, texture and inorganic composition of soils are strongly affected by their parental material. Strontium and Rb are very mobile elements during weathering processes, derived mainly from leachable minerals such as feldspars and micas [48]. Their and other metals bioavailability in soils is largely dependent on the partition of the metals between the solid and solution phases, but also on adsorption–precipitation and desorption–dissolution reactions that regulate the removal of nutrients from, or release into, the soil solution. The transfer of metals from the solid to the solution is also affected by factors such as pH, redox potential, organic matter, soil texture and clay content [49,50,51]. In this sense, to shed some lights on the influence of soil texture and clay content in the Sr isotopic composition of wines from vineyards with different geological substrata, a particle size analysis was performed in soils derived from metasediments (Desejosa and Rio Pinhão Formations) and granites (Melgaço and Monção) (Table S3). The texture of the fine earth fraction of those soils is predominantly sandy loam, but at Melgaço granitic area the texture is loamy sand. Among the analysed sandy loam soils, the granitic area of Monção contains the highest percentage of sand and the lowest percentage of clay, whereas the soil from Rio Pinhão Formation exhibit the opposite behavior. Therefore, it could be admissible a decrease in the permeability and an increase in the water holding capacity in the following sequence: Melgaço’s granitic soil →Monção’s granitic soil → Desejosa Formation’s soil → Rio Pinhão Formation’s soil. The texture and inorganic composition of soils from Pinhão Formation and Seixo de Ansiães areas are comparable to those from Rio Pinhão Formation and Melgaço domains, respectively, as expected considering their petrographic similarities. These results seem to point to a correlation between the Sr uptake by vine roots and the texture and clay content of soils, rather than with the Sr isotopic ratios of whole rocks or bulk soils. In fact, wines produced with grape varieties from vineyards emplaced in the sandy loam soils of Rio Pinhão and Pinhão Formations tend to yield the highest Sr ratios, whereas those resulting from vineyards emplaced in the more permeable soils, from Desejosa Formation metasediments and granitic areas, generally show lower Sr isotopic ratios.

The higher rate of adsorption of metals in the clay fraction, rather than in the silt and sand fractions, can explain the highest Sr ratios in the soils and also an increased ability of supplying plant nutrients during a specific period [49,50,51]. In fact, the study carried out by Ma & Liu [52] on weathered granites, evidenced that the fine grain-sized fractions were relatively enriched in Rb and Sr, whereas the clay-sized materials were much richer in Rb than Sr. The same study also demonstrated that the chemical weathering processes induced a fractionation between Rb and common Sr, which ultimately led to an increase or decrease of Rb/Sr ratios in the weathering products and to a concomitant behavior in their ^87^Sr/^86^Sr values, since ^87^Sr would be mainly derived from the ^87^Rb decay [52]. However, the higher Sr ratios in the soils can also be due to the presence of minerals with high Rb/Sr (and ^87^Sr/^86^Sr) values, which are more resistant to chemical weathering [52,53]. In opposition, the lower Sr isotopic ratios in the soils can be a direct consequence of their sand-silt enriched texture, with lower water holding capacity and, therefore, more favorable to leaching losses. On the other hand, a particular situation was detected within wines produced from vineyards emplaced in soils derived from two-mica granites of Melgaço and Seixo de Ansiães areas. Their Sr isotopic signatures show remarkable differences, with that of Melgaço presenting much lower ^87^Sr/^86^Sr values than the wine (Table S2; Figure 3B). Both granites yield similar petrographic and isotopic features and, therefore, this condition points to an influence of factors other than the texture and clay content of soils. A possible explanation can be related to the different topographic contexts of Melgaço and Seixo de Ansiães areas and to the intensity of deformation affecting both massifs. Indeed, the vineyards from Melgaço area are implanted in a steep/moderate slope terraced valley, where the outcropping granite yields a strong penetrative foliation, whereas those from Seixo de Ansiães area are located in a plateau area with a less deformed granitic substratum, which naturally will slow down the runoff and water percolation through the root-soil interface in the rhizosphere, increasing the nutrient supply capacity of those soils [54].

This research was carried out with a limited number of samples but, in general, the gathered results agree with the studies carried out by several authors in wines from Italian producing regions (Tuscany, Basilicata, Prosecco and Cesanese di Olevano Romano) [17,18,24,47], and from Canada [55,56] attesting the importance of Sr isotopic analysis in wines and their use as a geological fingerprint for tracing geographical provenance, even if in an indirect way. Therefore, the findings of this study can be very helpful in the recognition of a new perspective of the use of Sr isotopes in wine geographical traceability, which allowed the distinction among wines from two distinct granitic areas in the sub region Melgaço and Monção, but also among those from three metasedimentary formations from Douro demarcated region. To validate these first results, additional Pb isotopic data for whole rocks and wines will be presented and discussed in an integrated way in the following item.

#### 3.2.2. Tracing Wine Origin Based on Pb Isotopic Composition

According to Mihaljevič et al. [57] the total lead content in wine has three major sources: (1) in soils formed by bedrock’s weathering, (2) in fertilizers, pesticides and substances released during food processing and (3) through the pollution of the environment [57]. Thus, considering the non-contaminated environment of the studied areas, their vines should present Pb isotopic compositions shifted to geogenic characteristics, fact that can be used to assess their authenticity and geographical origin [58,59].

Some recent studies stated that lead uptake in plants occurs mainly through the foliage and in a lesser extend through the root system [57,60]. Nevertheless, the texture and mineralogy of soils and underlying bedrocks have certainly a major role in the Pb bioavailable control [49,50]. Lead is a moderate mobile element during weathering processes [61], occurring in resistate phases (e.g., zircon and monazite) and adsorbed on clay and (hydr-) oxides of iron and manganese or occluded in their lattice [49,61]. Thus, the release of radiogenic Pb might be caused by the alteration of resistate phases or by desorption reactions and, its availability depends on various process and factors influencing soil adsorptive properties, as well as on plant features [51].

The values of Pb isotopic ratios in whole rocks and wines from vineyards of six different locations are presented in Tables S4 and S5. The determined Pb isotopic ratios are given in terms of the atomic ^206^Pb/^204^Pb, ^207^Pb/^204^Pb and ^208^Pb/^204^Pb ratios because ^204^Pb is the only stable non-radiogenic isotope of Pb [53]. In the studied areas, the granites’ ^206^Pb/^204^Pb and ^207^Pb/^204^Pb values are always higher (19.0647 ± 0.0007 to 19.2859 ± 0.0006 and 15.6922 ± 0.0006 to 15.6930 ± 0.0006, respectively) than those of metasedimentary rocks (18.3067 ± 0.0009 to 18.5879 ± 0.0009 and 15.6475 ± 0.0007 to 15.6797 ± 0.0008), but no distinction can be made with respect to their ^208^Pb/^204^Pb values (Table S4; Figure 3C,D). In these rocks, zircon is the main U-bearing mineral and, despite its higher abundance in the metasediments, those crystals from Variscan granites yield considerably higher uranium contents [28,32,33]. This fact can explain the aforementioned differences, since ^206^Pb and ^207^Pb result from the decay of ^238^U and ^235^U, respectively [53]. The radiogenic ^208^Pb results from the ^232^Th decay and, therefore, ^208^Pb/^204^Pb values in the studied rocks should be mainly controlled by the presence of monazite and other Th-bearing minerals [32,33].

In the studied areas, the wines’ ^206^Pb/^204^Pb, ^207^Pb/^204^Pb and ^208^Pb/^204^Pb values are within the range of: 18.4441 ± 0.0008 (Alvarinho–Monção) to 18.7847 ± 0.0008 (Alvarinho–Rio Pinhão Formation); 15.6244 ± 0.0009 (Touriga Franca–Pinhão Formation) to 15.6699 ± 0.0008 (Alvarinho–Rio Pinhão Formation); and 38.3963 ± 0.0021 (Alvarinho–Monção) to 38.8573 ± 0.0020 (Alvarinho–Rio Pinhão Formation), respectively. Furthermore, there are no significant isotopic differences within the two monovarietal wines (Touriga Franca and Touriga Nacional) produced from the vineyards of Quinta do Portal and Quinta do Confradeiro (Desejosa Formation) (Table S5; Figure 3C), but at Quinta da Abelheira vineyards (Rio Pinhão Formation) the Alvarinho wine yield slightly higher ^207^Pb/^204^Pb and ^208^Pb/^204^Pb values than the Touriga Nacional wine (Table S5; Figure 3D,E), which may reflect some local differences in the soil characteristics. The determined ^206^Pb/^204^Pb values are somewhat higher than the calculated average (ca. 17.94) for the results obtained by Almeida and Vasconcelos in wine Port, table and red fortified wines from Douro region [62,63]. Naturally, these results are distinct from the mean values of Pb isotopic ratios determined by Larcher et al. [64] in wines from several provinces of Italy: ^206^Pb/^204^Pb = 17.84 ± 0.32, ^207^Pb/^204^Pb =15.24 ± 0.31 and ^208^Pb/^204^Pb = 37.31 ± 0.52,54 and also from those determined by Epova et al. [59] in authentic Bordeaux wines: ^206^Pb/^204^Pb = 18.191 ± 0.146, ^207^Pb/^204^Pb = 15.621 ± 0.014 and ^208^Pb/^204^Pb = 38.207 ± 0.156.

The Pb isotopic ratios of both bedrock and wine samples were determined at the same uncertainty level, so their relation can be depicted in the diagrams of Figure 3D,E. In general, a consistent depletion in ^207^Pb/^204^Pb and ^208^Pb/^204^Pb values from whole rock samples to the respective wines stands out, that is extended to the ^206^Pb/^204^Pb values of granites and respective wines. However, this latter isotopic ratio increases from the metasediments to the respective wines. These isotopic discrepancies observed between geological substrata and their respective wines can be due to mineralogical and textural specificities of each bedrock and respective derived soils. In fact, Marchionni et al. [18,19] stated that, in lithologies with high mineralogical variability, such as in granites, it would be plausible a differentiated isotopic uptake by vine roots, since the leachable phases could yield isotopic ratios different from those of the bulk rocks [18]. In this specific situation, considering the slow weathering rate of zircon, which is the main source ^206^Pb and ^207^Pb in metasediments and granites, lower ^206^Pb/^204^Pb and ^207^Pb/^204^Pb values would be expected for their respective wines [48]. Therefore, a possible explanation for the differentiated behavior in wines produced from vineyards of metasedimentary soils, yielding higher ^206^Pb/^204^Pb values than the respective whole rock samples, would be the higher rate of adsorption of lead in their enriched clay fraction, enabling a concomitant isotopic uptake by vine roots [49,51].

In the variation diagrams of Figure 3D,E it is also noticeable that wines produced from vineyards emplaced in metasediments yield higher ^206^Pb/^204^Pb and ^208^Pb/^204^Pb values (18.7505 ± 0.0010 to 18.7847 ± 0.0008 and 38.6557 ± 0.0023 to 38.8573 ± 0.0020, respectively) than those from granitic areas (18.4441 ± 0.0008 to 18.5474 ± 0.0010 and 38.3963 ± 0.0021 to 38.5834 ± 0.0025, respectively), but they are indistinct with the respect to their ^207^Pb/^204^Pb values (Figure 3D,E). As previously pointed, the soil texture and clay content from vineyards of the two studied demarcated regions show a decrease in the permeability and an increase in the water holding capacity in the following sequence: Melgaço’s granitic loamy sand soil → Monção’s granitic sandy loam soil → Desejosa Formation’s sandy loam soil → Rio Pinhão and Pinhão Formations’ sandy loam soils. Therefore, the highest ^206^Pb/^204^Pb and ^208^Pb/^204^Pb values of those wines produced in vineyards emplaced in the less permeable soils, derived from metasediments, suggest, as for Sr isotopic results, a correlation between the Pb uptake by vine roots and texture and clay content of soils, rather than with the whole rock’s Pb isotopic ratios. However, within granitic areas, the Alvarinho wine from Melgaço yields higher ^206^Pb/^204^Pb, ^207^Pb/^204^Pb and ^208^Pb/^204^Pb values than that from Monção (Figure 3D,E). Considering the higher permeability of Melgaço’s two-mica granitic soil, a plausible explanation for this situation could be the presence of zircon grains with higher uranium contents than those of the biotite granite of Monção [65]. The radiation damage caused in zircon crystals is consequently increased, facilitating the loss of lead in the soil solution [53].

#### 3.2.3. Tracing Wine Origin Using Combined Sr and Pb Isotopic Parameters

In general, the achieved results for Pb and Sr isotopic systems show an identical behavior within the two studied geological environments and respective wines. However, the Desejosa Formation’s metasediments/soils and wines yield Sr isotopic ratios similar to those from both granites of Monção and Melgaço (Figure 3B), but distinctive Pb isotopic signatures, which are instead related to those of the other studied metasedimentary environments (Figure 3C–F). This situation can be explained by the higher mobility of Sr over Pb during the weathering processes [48,49] together with the nature of the soil derived from Desejosa Formation’s metasediments, which contains an intermediate percentage of clay (Table S3) that would be sufficient to favor a Pb uptake by vine roots, but not to avoid the leaching losses of Sr.

By combining wine and bedrock’s ^207^Pb/^206^Pb and ^208^Pb/^206^Pb signatures it is possible to obtain a more perceptible and reliable differentiation scheme to distinguish wines according to their geographical origin. In fact, two wine/bedrock series, with distinctive linear regressions, can be identified among the studied samples (Figure 3F), which agrees with the study carried out by Epova et al. [59]. Although developed with a limited number of samples, it is evidenced the potential of the use of Pb isotopic ratios in the assessment of the geographical provenance and authenticity of wines from granitic areas in the sub region Melgaço and Monção and also from metasedimentary formations of the Douro demarcated region. A remark should be made regarding the possibility of combining wine and bedrock/soil’s Sr and Pb isotopic signatures to obtain a practical and effective wine geographical discriminator. The attempt made in the diagram of the Figure 3G indicates that, in spite of some scattering in Sr and Pb isotopic ratios of metasediments and respective soils, and the limited number of wine and rock/soil samples from granitic areas, it would be possible to predict radiogenic trends for three different geological environments: Melgaço granite, Monção granite and metasedimentary formations of the Douro demarcated region. However, further studies are required to test the robustness of those differentiation schemes, namely the evaluation of the influence of different degrees of weathering in soils on wine’s Pb isotopic signatures, which would be, in principle, placed along their particular regression lines.

## 4. Conclusions

The present study represents the basis for the development of an authenticity and geographical origin assessment system applied to wines from the sub region Melgaço and Monção of the demarcated Vinho Verde region and also to those from the demarcated Douro region. This system requires that both varietal composition and geographical provenance are correctly determined.

The use of DNA-based markers has proven to be efficient in the discrimination of the varietal composition of wines, as long as a panel of molecular markers is previously established [37,38]. Microsatellite database of grapevine have been defined by the OIV as being suitable for grapevine identification, however they are not consistent when dealing with wine samples. The resource to smaller DNA sequences is necessary to guarantee successful varietal identification in wine samples, such as SNP markers. Nevertheless, the definition of a SNP panel is required for general implementation by the OIV, possibly based on previous sets [43]. HRM assays were designed for grapevine varietal identification, based on the SNPs detection in the *UFGT*, *F3H* and *LDOX* genes [9,38]. The described multidisciplinary approach based on HRM assays and multi-elemental data fulfils our objectives and allows an accurate identification of the targeted varieties.

In terms of geographical provenance, the potential of a discriminating method based on the use of selected Sr and Pb isotopic signatures has been demonstrated, combined with textural and clay content analysis, which enable to distinguish wines produced from vineyards implanted in soils derived from metasedimentary and granitic environments. Concomitantly, these isotopic studies have also emphasized the importance of soil’s texture and clay content role in Sr and Pb uptake by vine roots.

However, further detailed studies, involving a higher number of bedrock, soil and wine samples of vineyards from different locations and distinct winemaking process, are still required to improve the reliability of authenticity criteria for these wines. In the future, these studies could benefit the provenance and authenticity assessments for Alvarinho, Porto and other Portuguese wines, and therefore help to avoid fraud and adulteration.

## Figures and Tables

**Table 1:** Provenance and other details of collected wine, soil and bedrock samples, from selected Portuguese vineyards.

Demarcated Region	Vineyards	Coordinates	Grape Variety	Geology
Vinho Verde	Quinta da Torre de Bardo–Monção	42°01′01.3″ N 8°29′37.5″ W	Alvarinho	Biotite granite
Vinho Verde	Quinta de Muros Antigos–Melgaço	42°04′47.1″ N 8°19′20.1″ W	Alvarinho	Two-mica granite
Douro	Estrada da Capela–Seixo de Ansiães	41°11′03.13″ N 7°16′09.94″ W	Touriga Nacional	Two-mica granite
Douro	Quinta do Portal–Celeirós	41°14′27.65″ N 7°33′54.89″ W	Touriga Nacional	Metasediment from Desejosa Formation
Douro	Quinta do Portal–Celeirós	41°14′23.0″ N 7°34′03.3″ W	Moscatel Galego	Metasediment from Desejosa Formation
Douro	Quinta do Portal–Celeirós	41°14′26.3″ N 7°33′56.9″ W	Touriga Franca	Metasediment from Desejosa Formation
Douro	Quinta do Confradeiro–Celeirós	41°14′12.4″ N 7°34′20.3″ W	Touriga Nacional	Metasediment from Desejosa Formation
Douro	Quinta dos Muros–Alijó	41°15′16.0″ N 7°32′02.4″ W	Touriga Franca	Metasediment from Pinhão Formation
Douro	Quinta da Abelheira–Alijó	41°15′03.6″ N 7°31′07.4″ W	Alvarinho	Metasediment from Rio Pinhão Formation
Douro	Quinta da Abelheira–Alijó	41°15′04.9″ N 7°30′53.0″ W	Moscatel Galego	Metasediment from Rio Pinhão Formation
Douro	Quinta da Abelheira–Alijó	41°15′04.9″ N 7°30′52.7″ W	Touriga Nacional	Metasediment from Rio Pinhão Formation

## Data Availability

The datasets generated for this study are available on request to the corresponding author.

## Supplementary Materials

The following are available online at https://www.mdpi.com/article/10.3390/foods10051044/s1, Table S1. Oenological characteristics of wines. Table S2. ^87^Sr/^86^Sr ratios for wine, soil and bedrock samples from selected Portuguese vineyards. Table S3. Particle size distribution in soils from from selected Portuguese vineyards. Table S4. ^206^Pb/^204^Pb, ^207^Pb/^204^Pb and ^208^Pb/^204^Pb ratios for bedrock samples from selected Portuguese vineyards. Table S5. ^206^Pb/^204^Pb, ^207^Pb/^204^Pb and ^208^Pb/^204^Pb ratios for wine samples from selected Portuguese vineyards.

## References

[1] Epova E.N., Bérail S., Séby F., Vacchina V., Bareille G., Médina B., Sarthou L., Donard O.F. (2019). Strontium elemental and isotopic signatures of Bordeaux wines for authenticity and geographical origin assessment. Food Chem..

[2] Council Regulation (EC) (2006). No 509/2006 of 20 March 2006 on agricultural products and foodstuffs as traditional specialities guaranteed. Off. J. Eur. Union.

[3] Council Regulation (EC) (2006). No 510/2006 of 20 March 2006 on the protection of geographical indications and designations of origin for agricultural products and foodstuffs. Off. J. Eur. Union.

[4] Commission Regulation (EC) (2006). No. 1898/2006 of 14 December laying down detailed rules of implementation of Council Regulation (EC) No. 510/2006 on the protection of geographical indications and designations of origin for agricultural products and foodstuffs. Off. J. Eur. Union.

[5] Cardoso A.S., Alonso J., Rodrigues A.S., Araújo-Paredes C., Mendes S., Valín M.I. (2019). Agro-ecological terroir units in the North West Iberian Peninsula wine regions. Appl. Geogr..

[6] Pii Y., Zamboni A., Santo S.D., Pezzotti M., Varanini Z., Pandolfini T. (2017). Prospect on Ionomic Signatures for the Classification of Grapevine Berries According to Their Geographical Origin. Front. Plant Sci..

[7] Villano C., Lisanti M.T., Gambuti A., Vecchio R., Moio L., Frusciante L., Aversano R., Carputo D. (2017). Wine varietal authentication based on phenolics, volatiles and DNA markers: State of the art, perspectives and drawbacks. Food Control.

[8] Belda I., Zarraonaindia I., Perisin M., Palacios A., Acedo A. (2017). From Vineyard Soil to Wine Fermentation: Microbiome Ap-proximations to Explain the “terroir” Concept. Front. Microbiol..

[9] Pereira L., Gomes S., Castro C., Eiras-Dias J.E., Brazão J., Graça A., Fernandes J.R., Martins-Lopes P. (2017). High Resolution Melting (HRM) applied to wine authenticity. Food Chem..

[10] Baleiras-Couto M., Eiras-Dias J. (2006). Detection and identification of grape varieties in must and wine using nuclear and chloroplast microsatellite markers. Anal. Chim. Acta.

[11] Vignani R., Liò P., Scali M. (2019). How to integrate wet lab and bioinformatics procedures for wine DNA admixture analysis and compositional profiling: Case studies and perspectives. PLoS ONE.

[12] Pereira L., Guedes-Pinto H., Martins-Lopes P. (2011). An Enhanced Method for *Vitis vinifera* L. DNA Extraction from Wines. Am. J. Enol. Vitic..

[13] Boccacci P., Akkak A., Marinoni D.T., Gerbi V., Schneider A. (2012). Genetic traceability of Asti Spumante and Moscatod’Asti musts and wines using nuclear and chloroplast microsatellite markers. Eur. Food Res. Technol..

[14] Barrias S., Fernandes J.R., Eiras-Dias J.E., Brazão J., Martins-Lopes P. (2019). Label free DNA-based optical biosensor as a potential system for wine authenticity. Food Chem..

[15] Catalano V., Moreno-Sanz P., Lorenzi S., Grando M.S. (2016). Experimental Review of DNA-Based Methods for Wine Traceability and Development of a Single-Nucleotide Polymorphism (SNP) Genotyping Assay for Quantitative Varietal Authentication. J. Agric. Food Chem..

[16] González-Neves G., Favre G., Piccardo D., Gil G. (2015). Anthocyanin profile of young red wines of Tannat, Syrah and Merlot made using maceration enzymes and cold soak. Int. J. Food Sci. Technol..

[17] Tescione I., Marchionni S., Casalini M., Vignozzi N., Mattei M., Conticelli S. (2018). ^87^ Sr/^86^ Sr isotopes in grapes of different cultivars: A geochemical tool for geographic traceability of agriculture products. Food Chem..

[18] Marchionni S., Braschi E., Tommasini S., Bollati A., Cifelli F., Mulinacci N., Mattei M., Conticelli S. (2013). High-Precision ^87^Sr/^86^Sr Analyses in Wines and Their Use as a Geological Fingerprint for Tracing Geographic Provenance. J. Agric. Food Chem..

[19] Marchionni S., Buccianti A., Bollati A., Braschi E., Cifelli F., Molin P., Parotto M., Mattei M., Tommasini S., Conticelli S. (2016). Conservation of ^87^ Sr/^86^ Sr isotopic ratios during the winemaking processes of ’Red’ wines to validate their use as geographic tracer. Food Chem..

[20] Catarino S., Castro F., Brazão J., Moreira L., Pereira L., Fernandes J., Eiras-Dias J., Graça A., Martins-Lopes P. (2019). ^87^Sr/^86^Sr isotopic ratios in vineyard soils and varietal wines from Douro Valley. BIO Web Conf..

[21] Moreira C., de Pinho M., Curvelo-Garcia A.S., de Sousa R.B., Ricardo-da-Silva J.M., Catarino S. (2017). Evaluating nanofil-tration effect on wine ^87^Sr/^86^Sr isotopic ratio and the robustness of this geographical fingerprint. S. Afr. J. Enol. Vitic..

[22] Malheiro V.L.T. (2008). Assinatura Geoquímica de Elementos Maioritários, Vestigiais e Razão Isotópica ^87^Sr/^86^Sr na Rocha, solo, Videira e Vinho. Master’s Thesis.

[23] Di Paola-Naranjo R.D., Baroni M.V., Podio N.S., Rubinstein H.R., Fabani M.P., Badini R.G., Inga M., Ostera H.A., Cagnoni M., Gallegos E. (2011). Fingerprints for Main Varieties of Argentinean Wines: Terroir Differentiation by Inorganic, Organic, and Stable Isotopic Analyses Coupled to Chemometrics. J. Agric. Food Chem..

[24] Durante C., Bertacchini L., Bontempo L., Camin F., Manzini D., Lambertini P., Marchetti A., Paolini M. (2016). From soil to grape and wine: Variation of light and heavy elements isotope ratios. Food Chem..

[25] Christoph N., Baratossy G., Kubanović V., Kozina B., Roßmann A., Schlicht C., Voerkelius S. (2004). Possibilities and limitations of wine authentication using stable isotope ratio analysis and traceability. Part 2: Wines from Hungary, Croatia and other European countries. Mitt. Klosterneubg..

[26] Ministério da Agricultura e do Mar (2015). Portaria 152/2015. Diário da República, série 101—26 de Maio de 2015.

[27] Ministério da Agricultura e do Mar (2016). Portaria 333/2016. Diário da República, série 245—23 de Dezembro de 2016.

[28] Teixeira R.J.S., Neiva A.M.R., Silva P.B., Gomes M.E.P., Andersen T., Ramos J.M.F. (2011). Combined U-Pb geochronology and Lu-Hf isotope systematics by LAM-ICP-MS of zircons from granites and metasedimentary rocks of Carrazeda de Ansiães and Sabugal areas, Portugal, to Constrain Granite Sources. Lithos.

[29] Gomes M.E.P., Teixeira R.J.S., Neiva A.M.R., Corfu F. (2014). Geochemistry and geochronology of granitoids from Bemposta-Picote region, Northeastern Portugal. Geoquímica e geocronologia dos granitóides da região de Bemposta-Picote, Nordeste de Portugal. Comun. Geológicas.

[30] Sousa M.B. (1982). Litoestratigrafia e Estrutura do Complexo Xisto-GrauváquicoAnte-Ordovícico Grupo do Douro (Nordeste de Portugal). Ph.D. Thesis.

[31] Dias R., Ribeiro A., Coke C., Pereira E., Rodrigues J., Castro P., Moreira N., Rebelo J., Dias R., Araújo A., Terrinha P., Kullberg J.C. (2013). Evolução estrutural dos sectores setentrionais do Autóctone da Zona Centro-Ibérica. InGeologia de Portugal: Volume I—Geologia Pré-mesozóica de Portugal.

[32] Aires C.M.A. (2018). Petrofísica e Litogeoquímica de Formações do “Complexo Xisto-Grauváquico” (Grupo do Douro). Estudo do potencial do “Xisto” Para Exploração Como Pedra Natural. Ph.D. Thesis.

[33] Teixeira R., Neiva A., Gomes M., Corfu F., Cuesta A., Croudace I. (2012). The role of fractional crystallization in the genesis of early syn-D_3_, tin-mineralized Variscan two-mica granites from the Carrazeda de Ansiães area, northern Portugal. Lithos.

[34] Teixeira R.J.S., Neiva A.M.R., Gomes M.E.P., Corfu F., Cuesta A., Croudace I.W. (2021). The importance of sequential partial melting and fractional crystallization in the generation of syn-D3 Variscan two-mica granites from the Carrazeda de Ansiães area, northern Portugal. J. Iber. Geol..

[35] International Organization of Vine and Wine-OIV (2020). Compendium of International Methods of Analysis of Wines and Musts.

[36] Doyle J.J., Doyle J.L. (1990). Isolation of plant DNA from fresh tissue. Focus.

[37] Pereira L., Martins-Lopes P. (2015). *Vitis vinifera* L. Single-Nucleotide polymorphism detection with high-resolution melting analysis based on the UDP-Glucose:Flavonoid 3-*O*-Glucosyltransferase gene. J. Agric. Food Chem..

[38] Gomes S., Castro C., Barrias S., Pereira L., Jorge P., Fernandes J.R., Martins-Lopes P. (2018). Alternative SNP detection platforms, HRM and biosensors, for varietal identification in *Vitis vinifera* L. using *F3H* and *LDOX* genes. Sci. Rep..

[39] Costa M., Neiva A., Azevedo M., Corfu F. (2014). Distinct sources for syntectonic *Variscan granitoids*: Insights from the Aguiar da Beira region, Central Portugal. Lithos.

[40] Deniel C., Pin C. (2001). Single-stage method for the simultaneous isolation of lead and strontium from silicate samples for isotopic measurements. Anal. Chim. Acta.

[41] Thirlwall M. (2002). Multicollector ICP-MS analysis of Pb isotopes using a ^207^pb-^204^pb double spike demonstrates up to 400 ppm/amu systematic errors in Tl-normalization. Chem. Geol..

[42] Santos S., Oliveira M., Amorim A., van Asch B. (2014). A forensic perspective on the genetic identification of grapevine (*Vitis vinifera* L.) varieties using STR markers. Electrophoresis.

[43] Cabezas J.A., Ibáñez J., Lijavetzky D., Vélez D., Bravo G., Rodríguez V., Carreño I., Jermakow A.M., Carreño J., Ruiz-García L. (2011). A 48 SNP set for grapevine cultivar identification. BMC Plant Biol..

[44] Cardoso J.C., Bessa M.T., Marado M.B. (1973). Carta de solos de Portugal (1/1000000). Agron. Lusit..

[45] Kawasaki A., Oda H., Hirata T. (2002). Determination of strontium isotope ratio of brown rice for estimating its provenance. Soil Sci. Plant Nutr..

[46] Geanã I., Sandru C., Stanciu V., Roxana I. (2017). Elemental Profile and ^87^Sr/^86^Sr Isotope Ratio as Fingerprints for Geographical Traceability of Wines: An Approach on Romanian Wines. Food Anal. Methods.

[47] Petrini R., Sansone L., Slejko F., Buccianti A., Marcuzzo P., Tomasi D. (2015). The ^87^Sr/^86^Sr strontium isotopic systematics applied to *Glera vineyards*: A tracer for the geographical origin of the Prosecco. Food Chem..

[48] Middelburg J.J., van der Weijden C.H., Woittiez J.R. (1988). Chemical processes affecting the mobility of major, minor and trace elements during weathering of granitic rocks. Chem. Geol..

[49] Rieuwerts J.S., Thornton I., Farago M.E., Ashmore M.R. (1998). Factors influencing metal bioavailability in soils: Preliminary investigations for the development of a critical loads approach for metals. Chem. Spec. Bioavailab..

[50] Comerford N.B., Bassiri-Rad H. (2005). Soil factors affecting nutrient bioavailability. Nutrient Acquisition by Plants. An Ecological Perspective. Ecological Studies. Analysis and Synthesis.

[51] Burger A., Lichtscheidl I. (2019). Strontium in the environment: Review about reactions of plants towards stable and radioactive strontium isotopes. Sci. Total Environ..

[52] Ma Y., Liu C. (2001). Sr isotope evolution during chemical weathering of granites. Sci. China Ser. D Earth Sci..

[53] Faure G., Mensing T.M. (2005). Isotopes—Principles Applications.

[54] Katoh M., Murase J., Hayashi M., Matsuya K., Kimura M. (2004). Nutrient leaching from the plow layer by water percolation and accumulation in the subsoil in an irrigated paddy field. Soil Sci. Plant Nutr..

[55] Vinciguerra V., Stevenson R., Pedneault K., Poirier A., Hélie J.F., Widory D. (2015). Strontium isotope characterization of wines from the Quebec (Canada) Terroir. Procedia Earth Planet. Sci..

[56] Vinciguerra V., Stevenson R., Pedneault K., Poirier A., Hélie J.-F., Widory D. (2016). Strontium isotope characterization of wines from Quebec, Canada. Food Chem..

[57] Mihaljevič M., Ettler V., Sebek O., Strnad L., Chrastný V. (2006). Lead isotopic signatures of wine and vineyard soils-tracers of lead origin. J. Geochem. Explor..

[58] Margui E., Iglesias M., Queralt I., Hidalgo M. (2006). Lead isotope ratio measurements by ICP-QMS to identify metal accumulation in vegetation specimens growing in mining environments. Sci. Total Environ..

[59] Epova E.N., Bérail S., Séby F., Barre J.P., Vacchina V., Médina B., Sarthou L., Donard O.F. (2020). Potential of lead elemental and isotopic signatures for authenticity and geographical origin of Bordeaux wines. Food Chem..

[60] Lim M.P., McBride M.B. (2015). Arsenic and lead uptake by Brassicas grown on an old orchard site. J. Hazard. Mater..

[61] Peng B., Rate A., Song Z., Yu C., Tang X., Xie S., Tu X., Tan C. (2014). Geochemistry of major and trace elements and Pb–Sr isotopes of a weathering profile developed on the Lower Cambrian black shales in central Hunan, China. Appl. Geochem..

[62] Almeida C.M.R., Vasconcelos T.S.D. (1999). Determination of lead isotope ratios in port wine by inductively coupled plasma mass spectrometry after pre-treatment by UV-irradiation. Anal. Chim..

[63] Almeida C.M.R., Vasconcelos T.S.D. (2003). Lead contamination in Portuguese red wines from the Douro region: From the vineyard to the final product. J. Agric. Food Chem..

[64] Larcher R., Nicolini G., Pangrazzi P. (2003). Isotope ratios of lead in Italian wines by inductively coupled plasma mass spectrometry. J. Agric. Food Chem..

[65] Dias G., Leterrier J., Mendes A., Simões P., Bertrand J. (1998). U-Pb zircon and monazite geochronology of post-collisional Hercynian granitoids from the Central Iberian Zone (Northern Portugal). Lithos.

