# Supplementary material for: A Multidisciplinary Fingerprinting Approach for Authenticity and Geographical Traceability of Portuguese Wines
Source: Foods. 2021 May 11;10(5):1044. doi: 10.3390/foods10051044 (PMC8150803; doi:10.3390/foods10051044)
Supplement: Supplementary file 1 [file foods-10-01044-s001.zip › foods-1181713-supplementary.pdf]

Supplementary data

Table S1. $^{87}Sr/^{86}Sr$ ratios for wine, soil and rock samples from selected Portuguese vineyards

Demarcated region	Vineyards	Grape variety	Geology	Wine		Soil		Rock	
				$^{87}Sr/^{86}Sr$	2 σ	$^{87}Sr/^{86}Sr$	2 σ	$^{87}Sr/^{86}Sr$	2 σ
Vinho Verde	Quinta da Torre de Bardo - Monção	Alvarinho	Biotite granite	0.719349	0.000022	0.753405	0.000020	0.748155	0.000025
Vinho Verde	Quinta de Muros Antigos - Melgaço	Alvarinho	Two-mica granite	0.713981	0.000024	0.779035	0.000019	0.783222	0.000020
Douro	Estrada da Capela - Seixo de Ansiães	Touriga Nacional	Two-mica granite	0.719199	0.000046	0.779225	0.000034	0.770011	0.000028
Douro	Quinta do Portal - Celeirós	Touriga Nacional	Metasediment from Desejosa Formation	0.718108	0.000030	0.740815	0.000031	0.739819	0.000036
Douro	Quinta do Portal - Celeirós	Moscatel Galego	Metasediment from Desejosa Formation	0.715493	0.000016	0.757269	0.000026	0.766515	0.000025
Douro	Quinta do Portal - Celeirós	Touriga Franca	Metasediment from Desejosa Formation	0.716454	0.000019	0.751334	0.000018	0.727938	0.000020
Douro	Quinta do Confradeiro - Celeirós	Touriga Nacional	Metasediment from Desejosa Formation	0.719856	0.000020	0.757056	0.000020	0.764387	0.000015
Douro	Quinta dos Muros - Alijó	Touriga Franca	Metasediment from Pinhão Formation	0.721478	0.000017	0.739361	0.000024	0.734324	0.000022
Douro	Quinta da Abelheira - Alijó	Alvarinho	Metasediment from Rio Pinhão Formation	0.719344	0.000014	0.728944	0.000020	0.728796	0.000019
Douro	Quinta da Abelheira - Alijó	Moscatel Galego	Metasediment from Rio Pinhão Formation	0.720706	0.000017	0.752811	0.000023	0.739859	0.000022

Douro	Quinta da Abelheira - Alijó	Touriga Nacional	Metasediment from Rio Pinhão Formation	0.721116	0.000019	0.751095	0.000029	0.755171	0.000015

Table S2. Particle size distribution in soils from from selected Portuguese vineyards

Demarcated region	Vineyards	Grape variety	Geology	Clay (g/kg)	Silt (g/kg)	Fine sand (g/kg)	Coarse sand (g/kg)	Soil texture
				(< 0.002 mm)	(0.002 – 0.02 mm)	(0.02 – 0.2 mm)	(0.2 – 2 mm)	
Vinho Verde	Quinta da Torre de Bardo - Monção	Alvarinho	Biotite granite	97	129	213	562	Sandy loam
Vinho Verde	Quinta de Muros Antigos - Melgaço	Alvarinho	Two-mica granite	77	110	191	622	Loamy sand
Douro	Quinta do Confradeiro - Celeirós	Touriga Nacional	Metasediment from Desejosa Formation	108	165	316	412	Sandy loam
Douro	Quinta da Abelheira - Alijó	Touriga Nacional	Metasediment from Rio Pinhão Formation	112	231	399	257	Sandy loam

Table S3. $^{206}Pb/^{204}Pb$, $^{207}Pb/^{204}Pb$ and $^{208}Pb/^{204}Pb$ ratios for bedrock samples from selected Portuguese vineyards

Demarcated region	Vineyards	Grape variety	Geology	Rock					
				$^{206}Pb/^{204}Pb$	2 σ	$^{207}Pb/^{204}Pb$	2 σ	$^{208}Pb/^{204}Pb$	2 σ
Vinho Verde	Quinta da Torre de Bardo - Monção	Alvarinho	Biotite granite	19.2859	0.0006	15.6930	0.0006	39.0449	0.0018
Vinho Verde	Quinta de Muros Antigos - Melgaço	Alvarinho	Two-mica granite	19.0647	0.0007	15.6922	0.0006	38.7425	0.0018
Douro	Quinta do Portal - Celeirós	Touriga Franca	Metasediment from Desejosa Formation	18.3067	0.0009	15.6797	0.0008	39.1033	0.0022
Douro	Quinta do Confradeiro - Celeirós	Touriga Nacional	Metasediment from Desejosa Formation	18.3871	0.0008	15.6687	0.0008	39.0905	0.0023
Douro	Quinta dos Muros - Alijó	Touriga Franca	Metasediment from Pinhão Formation	18.4169	0.0008	15.6475	0.0007	38.8575	0.0020
Douro	Quinta da Abelheira - Alijó	Alvarinho	Metasediment from Rio Pinhão Formation	18.5655	0.0010	15.6653	0.0009	39.0449	0.0025
Douro	Quinta da Abelheira - Alijó	Touriga Nacional	Metasediment from Rio Pinhão Formation	18.5879	0.0009	15.6570	0.0009	39.2372	0.0026

Table S4. $^{206}Pb/^{204}Pb$, $^{207}Pb/^{204}Pb$ and $^{208}Pb/^{204}Pb$ ratios for wine samples from selected Portuguese vineyards

**Demarcated region**	**Vineyards**	**Grape variety**	**Geology**	**Wine**					
				**$^{206}Pb/^{204}Pb$**	**2 σ**	**$^{207}Pb/^{204}Pb$**	**2 σ**	**$^{208}Pb/^{204}Pb$**	**2 σ**
Vinho Verde	Quinta da Torre de Bardo - Monção	Alvarinho	Biotite granite	18.4441	0.0008	15.6264	0.0007	38.3963	0.0021
Vinho Verde	Quinta de Muros Antigos - Melgaço	Alvarinho	Two-mica granite	18.5474	0.0010	15.6601	0.0010	38.5834	0.0025
Douro	Quinta do Portal - Celeirós	Touriga Franca	Metasediment from Desejosa Formation	18.7577	0.0012	15.6357	0.0011	38.6669	0.0029
Douro	Quinta do Confradeiro - Celeirós	Touriga Nacional	Metasediment from Desejosa Formation	18.7834	0.0010	15.6290	0.0009	38.6760	0.0023
Douro	Quinta dos Muros - Alijó	Touriga Franca	Metasediment from Pinhão Formation	18.7825	0.0010	15.6244	0.0009	38.6557	0.0023
Douro	Quinta da Abelheira - Alijó	Alvarinho	Metasediment from Rio Pinhão Formation	18.7847	0.0008	15.6699	0.0008	38.8573	0.0020
Douro	Quinta da Abelheira - Alijó	Touriga Nacional	Metasediment from Rio Pinhão Formation	18.7505	0.0010	15.6310	0.0010	38.6888	0.0026
